# Can Non-Physician Providers Use Ultrasound to Aid in Establishing Peripheral IV Access in Patients Who are Difficult to Cannulate? A Scoping Review

**DOI:** 10.1017/S1049023X22000796

**Published:** 2022-08

**Authors:** Samuel O. Burton, Jake K. Donovan, Samuel L. Jones, Benjamin N. Meadley

**Affiliations:** 1. Ambulance Victoria, Doncaster, Victoria, Australia; 2. Department of Paramedicine, Monash University, Frankston, Victoria, Australia

**Keywords:** IV access, non-physician, peripheral venous access, POCUS, ultrasound

## Abstract

**Introduction:**

Non-physician performed point-of-care ultrasound (POCUS) is emerging as a diagnostic adjunct with the potential to enhance current practice. The scope of POCUS utility is broad and well-established in-hospital, yet limited research has occurred in the out-of-hospital environment. Many physician-based studies expound the value of POCUS in the acute setting as a therapeutic and diagnostic tool. This study utilized a scoping review methodology to map the literature pertaining to non-physician use of POCUS to improve success of peripheral intravenous access (PIVA), especially in patients predicted to be difficult to cannulate.

**Methods:**

Ovid MEDLINE, CINAHL Plus, EMBASE, and PubMed were searched from January 1, 1990 through April 15, 2021. A thorough search of the grey literature and reference lists of relevant articles was also performed to identify additional studies. Articles were included if they examined non-physician utilization of ultrasound-guided PIVA (USGPIVA) for patients anticipated to be difficult to cannulate.

**Results:**

A total of 158 articles were identified. A total of 16 articles met the inclusion criteria. The majority of participants had varied experience with ultrasound, making accurate comparison difficult. Training and education were non-standardized, as was the approach to determining difficult intravenous access (DIVA). Despite this, the majority of the studies demonstrated high first attempt and overall success rates for PIVA performed by non-physicians.

**Conclusion:**

Non-physician USGPIVA appears to be a superior method for PIVA when difficulty is anticipated. Additional benefits include reduced requirement for central venous catheter (CVC) or intraosseous (IO) needle placement. Paramedics, nurses, and emergency department (ED) technicians are able to achieve competence in this skill with relatively little training. Further research is required to explore the utility of this practice in the out-of-hospital environment.

## Introduction

Peripheral intravenous (IV) catheterization is one of the most commonly performed procedures by non-physicians in both the emergency department (ED) and out-of-hospital environment.^1–3^ Presently, most providers employ the conventional peripheral intravenous access (PIVA) method, with difficulty often encountered in both anatomically challenging and critically unwell patients.^1,2^ The overall failure of PIVA is reportedly from 10% through 40% in EDs, intensive care units, and in the out-of-hospital setting.^4^ Failure of the first attempt has been reported to occur in up to 67% of patients requiring subsequent or multiple punctures.^5^

Ultrasound-guided PIVA (USGPIVA) occurs routinely in the hospital setting when difficulty is either predicted or encountered. In-hospital clinical studies indicate that ultrasound guidance significantly improves the overall success rate of PIVA and reduces the number of punctures required, time to successful PIVA, physician intervention, and rate of central venous catheter (CVC) insertion.^4,6–10^ The implications of failed or inadequate PIVA are varied, often resulting in escalation to a more senior clinician and potentially an alternative vascular access strategy.^9,10^ Alternative vascular access is often achieved through the insertion of a CVC in-hospital and intraosseous (IO) access in the out-of-hospital environment.^9,11^ Both CVC and IO insertion expose the patient to a range of additional risks that could be avoided with successful PIVA, including bloodstream infection fat emboli, pneumothorax, large artery puncture, impaired flow rates, and osteomyelitis.^9,12^ These are undesirable risks for patients where PIVA is less-invasive and sufficiently meets care requirements.

Determining patients at risk for difficult intravenous access (DIVA) has historically relied on the clinicians’ experience and clinical gestalt. Patient characteristics associated with difficult PIVA have been identified and developed into externally validated assessment tools that are predictive of adult patients at risk of DIVA, including the Adult – Difficult Intravenous Access (A-DIVA) scale.^13^

Increased availability and portability of handheld ultrasound devices has made this practice a realistic consideration for the out-of-hospital setting. Paired with a predictive A-DIVA scale, the adoption of point-of-care ultrasound (POCUS) can significantly improve first attempt success, reduce the occurrence of multiple punctures, and reduce overall time to successful PIVA.^5,13^ The efficacy of USGPIVA in-hospital is firmly established when performed by physicians,^14^ but such data are not available for non-physicians, particularly in the out-of-hospital environment. This paper aimed to identify the available evidence for the utility of POCUS in anticipated difficult PIVA by non-physicians.

## Methods

The authors searched, compiled, and reviewed the available literature relating to paramedic use of POCUS to establish IV access in the out-of-hospital environment. Preliminary searches of EMBASE (Elsevier; Amsterdam, Netherlands) and Ovid (Ovid Technologies; New York, New York USA) databases revealed limited literature on the subject. The study used a scoping review methodology in order to develop a specific research question. In alignment with established scoping review procedure, the study included peer and non-peer-reviewed articles in addition to grey literature. This study employed the six-stage methodology as described by Levac, et al.^15^

The research question was identified as: “Can non-physicians use ultrasound to aid in establishing IV access in patients who are difficult to cannulate?” After initial review of the literature, the authors decided upon this question as it was felt to both capture a range of articles while remaining focused enough to facilitate a search strategy.

A preliminary search of online databases EMBASE and Ovid was conducted to identify literature relevant to the topic. Keywords and index terms from the retrieved articles were analyzed and then included in the second search. The online databases Ovid MEDLINE (US National Library of Medicine, National Institutes of Health; Bethesda, Maryland USA); EMBASE; PubMed (National Center for Biotechnology Information, National Institutes of Health; Bethesda, Maryland USA); and CINAHL Plus (EBSCO Information Services; Ipswich, Massachusetts USA) were then searched from January 1, 1990 through April 15, 2021 including the identified terms, Medical Subject Headings (MeSH terms), and keywords relevant to out-of-hospital care, paramedics, and ultrasound-guided peripheral IV cannulation. A thorough search of the grey literature and reference lists of relevant papers was also appraised to identify additional articles. The search strategy consisted of Boolean terms and operators within the population/concept/context (PCC) format (Table 1).

**Table 1.** Summary of Population/Concept/Context (PCC) Search Terms

PCC Element	Definition	Search Term
Population	Participant features ▪ Adults >18 years ▪ Anticipated difficult IV access	
Concept	Interventions/outcomes ▪ Utilization of US device to achieve peripheral IV access	▪ IV access.mp ▪ Intravenous access.mp ▪ Peripheral venous access.mp ▪ Vascular access.mp ▪ Catheterization, peripheral/ ▪ Ultrasound-guided.mp ▪ Ultrasound-guided procedure.mp ▪ Venous ultrasound.mp ▪ Ultraso*.mp ▪ Vascular ultraso*.mp ▪ POCUS.mp
Context	Details of setting ▪ Any setting ▪ Non-physician providers	▪ Out of hospital.tw ▪ Emergency department.tw ▪ Emergency medical services.sh ▪ Emergency medical technicians.sh ▪ HEMS.tw ▪ Ambulance.tw ▪ Ambulances.sh ▪ EMS.tw ▪ EMT.tw ▪ Emergency services.tw ▪ First responder*.tw ▪ Pre hospital.tw ▪ Pre-hospital.tw ▪ Paramedic.tw ▪ Non-physician.tw

Abbreviations: IV, intravenous; POCUS, point-of-care ultrasound; HEMS, helicopter Emergency Medical Services; EMS, Emergency Medical Services; EMT, emergency medical technician.

Eligibility was defined by: (1) non-physicians in any setting utilizing POCUS to guide peripheral venous cannulation, and (2) published from January 1, 1990 through April 15, 2021. The time period was determined after preliminary search produced no studies of relevance prior to 1990. In addition, small and portable POCUS devices are technologically modern and have only been adopted into medical practice in more recent times.^16^ Studies were excluded if they were performed by physicians, literature reviews, not published in English, based on opinion or commentary, and if they were based on training or simulation.

**Figure 1.**
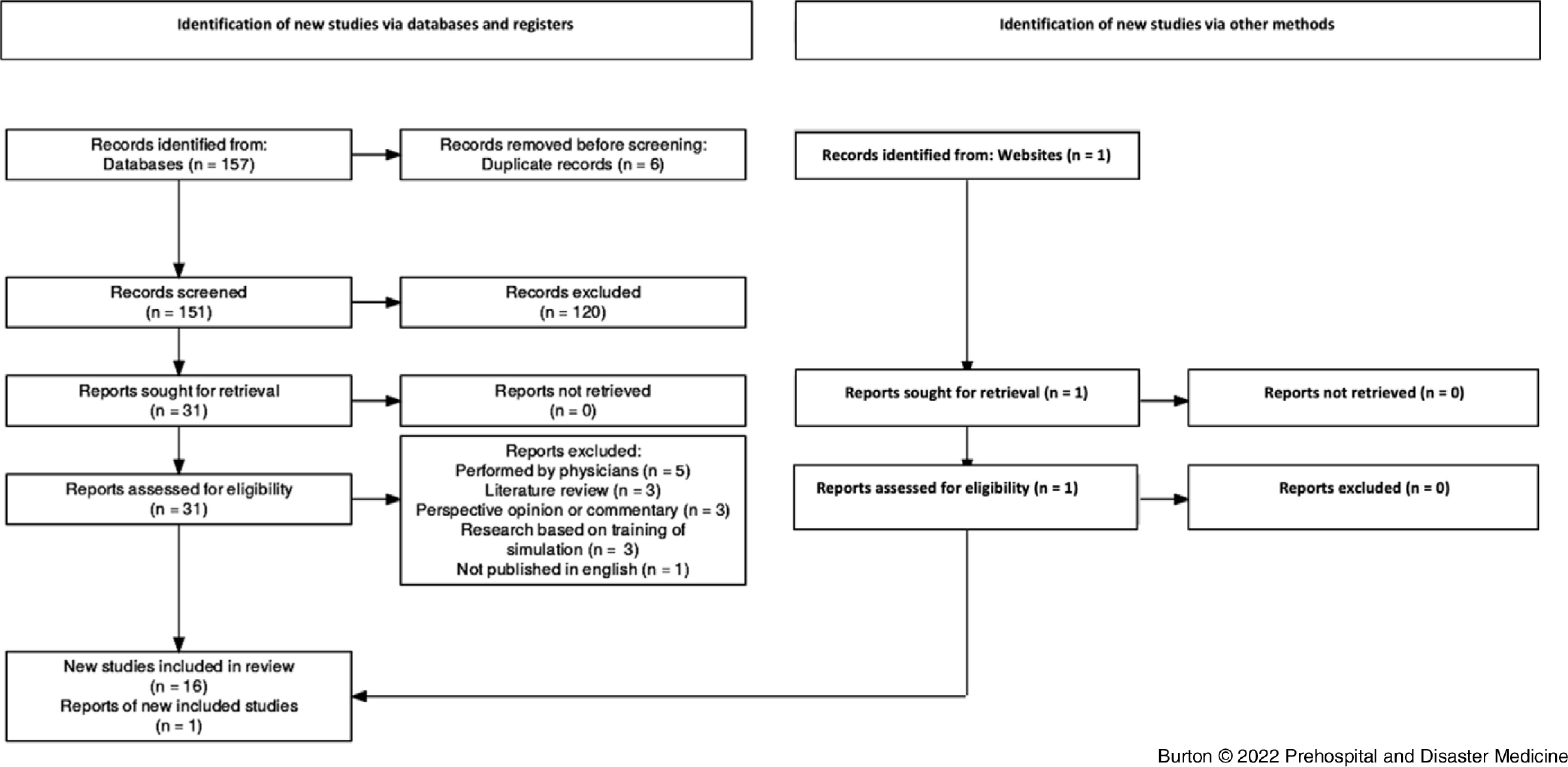
Flow Diagram Showing Identification of Studies Evaluating Non-Physician USGPIVA for Patients Anticipated to be Difficult. Abbreviation: USGPIVA, ultrasound-guided peripheral intravenous access.

The databases were searched by one author (SB). Duplicates were then removed, followed by eligibility screening of titles and abstracts by three authors (SB, BM, and JD). The full texts of the remaining articles were then sourced and reviewed (Figure 1). A “descriptive analytical” approach was used to extract relevant data from each of the studies. This has then been collated into table form to provide an overview of the 17 articles selected for inclusion. Key information was identified and charted as per common analytical framework.^17^

A total of 16 studies were included in the review, comprising eight prospective observational studies, three retrospective observational studies, two randomized control trials, one prospective non-blinded randomized control trial, one retrospective cohort study, and one prospective, randomized, comparative evaluation. The summary results are depicted below in addition to a summary in Table 2.^4,5,9,10,18–29^

**Table 2.** Study Characteristics and Educational Approach

Study	Participants	Study Design	Aim	Setting	Protocol Details	Education
Acuña, et al 2020^19^	Nurses Paramedics	Prospective Observational Study	Evaluate performance of a handheld US device for difficult PIVA as performed by nurses/paramedics in the ED	ED – United States	Discretionary assessment of difficulty by operator based on failed attempts or perceived difficulty. Patients deemed difficult were enrolled and assigned either USGPIVA or SOC. Success defined as catheter visualized within the vessel and able to be flushed easily.	Device – Philips Lumify. 8-hour educational session (lecture and didactic education). Familiarization with POCUS device and how to optimize image quality.
Ault, et al 2015^23^	Nurses	Prospective Observational Study	To determine the number of US-guided IV placements required for a nurse to develop proficiency and consistency	Medical Procedure Center – United States	Difficulty assessed by operators if there was a lack of a palpable or visible vessel or if the patient had a history of requiring US-guided IV access or central venous access. Proficiency determined by 10 successful supervised attempts and proficiency score of 4 or 5 for 3 consecutive attempts.	Device – Sonosite M-Turbo. 3-phase educational program including 1:1 didactic session, demonstration of proficiency on phantom model, and supervised attempts on live patients.
Bahl, et al 2016^20^	Nurses	Prospective, Non-Blinded, Randomized Control Trial	Investigated the outcomes associated with nurse performed US- guided IV access when compared to landmark approach on difficult vascular access patients	ED – United States	Patients presenting to ED were randomized to 1 of 2 cannulation techniques. Either USGPIVA or SOC (landmark method). Success was determined by the extraction of 5ml of non-pulsatile blood or flush of 5ml normal saline. Developed robust inclusion criteria to select DIVA patients.	Device – Sonosite M-Turbo. Participants attended a 1.5-hour didactic educational session, followed by hands on familiarization. Certification was provided upon 10 successful IV placements.
Duran-Gehring, et al 2016^24^	ED Technicians	Retrospective Review of Prospectively Collected Data	To determine the success and complication rates of ED technicians performing US-guided peripheral IV placement	ED – United States	None of the participants had prior US experience. An algorithm was developed to predict difficult IV with physician input. Patients were then potentially enrolled to receive up to 3 US-guided IV attempts by the participants with success, failure, and complication rates recorded.	Device – Sonosite M-Turbo. 18 emergency technicians (paramedics) enlisted to participate. 3-phase educational program beginning with training, demonstration of competence, and then clinical application.
McCarthy, et al 2016^25^	ED Technicians	Randomized Control Trial with a 2-Group Parallel Design	To determine the superior method of IV placement in patients with varying levels of difficulty	ED – United States	Patients enrolled were sorted into easy access, moderately difficult, and difficult access groups. Enrolled patients were then randomized and assigned to either USGPIVA or SOC. If first attempt failed, the patient was then randomized a second time to a procedure.	Device – Sonosite M-Turbo or Zonare Ultra. All of the participants were familiar and proficient with the procedure as part of their existing practice.
Oliveira, et al 2016^26^	Nurses Military Corpsmen	Prospective Observational Study	To develop a program to train nurses, corpsmen, and physicians in US-guided IV placement and assess the degree of success in outcomes	Military Hospital – United States	Two of the nurse participants had prior US experience. The program developed was not defined in this study.	Device – Sonosite M-Turbo. 8 nurses and 8 corpsmen participated in the study. Nurses and corpsmen were required to attend one training session, comprised of a 30-minute didactic session, and complete 3 supervised US-guided IV placements.
Price, et al 2019^22^	Nurses ED Technicians	Prospective, Randomized, Comparative Evaluation Study	To determine if US-guided IV placement first attempt success is improved with double tourniquet technique	Tertiary Care Hospital ED – United States	Patients had to have had one failed blind attempt at IV placement, >18 years old, and predicted to be difficult to be enrolled in the study. Patients enrolled were then randomized to either single or double tourniquet technique followed by USGPIVA.	Device – Sonosite X-Porte. All participants had minimum 1 year experience with the procedure and no education was offered prior to commencement of the study.
Resnick, et al 2008^27^	Nurses	Prospective, Randomized, Comparison Study	To compare the practice of no skin marking versus no skin marking when performing US-guided PIVA	ED – United States	Participants were categorized by the number of USGPIVA attempts and experience they previously had. Patients were enrolled and randomized to either no skin marking or skin marking approach. UGPIVA was then attempted either with or without skin marking.	Device – Sonosite Titan L38. Nurses were given a 2-hour educational session including simulated practice on phantom models.
Salleras-Duran, et al 2016^4^	Nurses	Descriptive, Observational Study	To examine the success of US-guided IV placement in patients predicted to be difficult	ED – Spain	All patients requiring peripheral IV, >18 years old, and met requirements for US-guided IV placement were included. Patients indicated for US-guided IV placement were those determined difficult by the nurse operator using a 10-point Likert scale. Nurses recorded variables after each procedure for evaluation.	Device – N/A. Participants completed a 20-huor training course covering US basic concepts and simulated practice.
Schoenfeld, et al 2011^28^	ED Technicians	Prospective Observational Study	To assess the success of ED technicians when placing US-guided peripheral IV catheters	ED – United States	At least two attempts at traditional IV placement had to have occurred, and/or patients with established history of difficult access requiring alternative intervention to be included in the study. Technicians completed a survey at the completion of each survey, documenting a range of variables.	Device – Sonosite M-Turbo. 19 ED technicians participated in the study. Participants completed a 2-hour training session that was didactic and hands-on.
Shokohi, et al 2013^9^	ED Technicians	Retrospective Cohort Study	To assess whether the introduction of US-guided IV access program in the ED resulted in less CVC use	ED – United States	Study period was 6 years. Investigators observed the rate of CVC placement after the implementation of an US-guided IV placement program.	Device – N/A. Technicians were provided with 2-hour educational session comprised of both didactic and hands-on learning.
Skulec, et al 2020^5^	Paramedics	Controlled, Prospective, Randomized, Non-Blinded Clinical Study	To compare two different approaches of US-guided IV placement and the landmark method of IV placement by paramedics	Out-of-Hospital – Czech Republic	5 paramedics participated in the study. Patients were included if they were conscious and indicated for prehospital IV placement. Enrolled patients were then randomized in a predefined 1:1:1 ratio to IV insertion fully controlled by US guidance, IV insertion partially controlled by US guidance (target vein identification only), or landmark method.	Device – GE Vscan Dual Probe. Paramedics were naïve to US prior to commencement of the study. Paramedics attended a 1-day emergency POCUS course for beginners that comprised of both hands-on and didactic education sessions.
Stolz, et al 2016^18^	Nurses Paramedics	Prospective Observational Study	To determine how many attempts were required to achieve proficiency with US-guided IV placement in nurses and paramedics	ED – United States	All participants were previously naïve to the USGPIVA placement procedure but proficient in traditional PIVA. Interested nurses or paramedic could electively enroll in the training to participate in the program. Participants were required to complete a survey after each procedure, documenting variables for collection.	Device – Mindray M7 and Ultrasonix SonixTouch. 33 participants were included in the study. Each were provided with 2-hours of training including didactic and hands components.
Vinograd, et al 2018^29^	Nurses	Prospective Observational Study	To examine the success, complications, and longevity of US-guided IVs placed in a pediatric ED	ED – United States	Patients were included after multiple failed blind attempts, a history of difficulty, educational purposes, and patient or family request. Participants completed a survey after each procedure and documented key information.	Device – N/A. 24 nurses participated in this study. Nurse participants were provided with 4-hour training session including didactic and hands-on components.
Weiner, et al 2013^10^	Nurses	Two-Site, Prospective, Non-Blinded, Pilot Study	To determine if trained emergency nurses can place US-guided IVs and subsequently require less physician intervention	ED – United States	Patients were enrolled in a convenience sample and assigned to either SOC or US-guided IV arm. Patients were included if they were adults, indicated for IV access, and were predicted to be difficult.	Device – Sonosite M-Turbo and Zonarae z.one Ultra Convertible Ultrasound System. Each participant was provided with 2-hour training session including didactic and hands-on components.
Miles, et al 2012^21^	Nurses	Prospective, Multicenter, Pilot Study	To evaluate the success of program implemented to facilitate nurse led US-guided PIVA in the ED	ED – United States	Patients were eligible for inclusion if they either had two failed blind attempts or reported a history of DIVA. Consenting patients were assigned to have either US-guided IV access or SOC.	Device – Sonosite MicroMaxx Portable. Participants received 8-hour tutorial from experienced emergency physician including didactic and hands-on elements.

Abbreviations: ED, emergency department; US, ultrasound; PIVA, peripheral intravenous access; IV, intravenous; CVC, central venous catheter; USGPIVA, ultrasound-guided PIVA; SOC, standard of care; DIVA, difficult intravenous access; POCUS, point-of-care ultrasound.

## Results

The initial search generated 151 articles after six duplicates were removed. The titles and abstracts of the relevant articles were then screened for inclusion and 120 were excluded as per the study protocol (Figure 1). One additional study was identified through a grey literature search of Google Scholar (Google Inc.; Mountain View, California USA) and added to the review. The final review included a total of 16 studies, the characteristics of which are presented in Table 2.

### Participants

The participant population varied between nurses, paramedics, and emergency technicians. Experience was also varied with some operators proficient with USGPIVA placement and others naïve to POCUS. Most studies included a combined cohort of clinicians with a broad range of clinical experience. Only one study described a paramedic-only cohort and was solely based in the out-of-hospital setting.

### Scan Protocol

Three out of the 16 studies examined paramedic application of USGPIVA, two within the ED and one out-of-hospital.^5,18,19^ Each study measured different outcomes making it difficult to compare and evaluate performance. Acuña, et al aimed to evaluate the performance of a handheld POCUS device as used by paramedics and nurses to perform USGPIVA in the ED. The study enrolled a cohort of 483 participants and reported first attempt success of 84% using a discretionary approach to determine difficulty.^19^ The only out-of-hospital study was a randomized, control trial performed by Skulec, et al and evaluated paramedics’ success performing USGPIVA with a handheld POCUS device.^5^ Only five paramedics participated in the study, however, 300 patients were enrolled and randomized equally into three groups. Group A received USGPIVA access under complete ultrasound guidance where the catheter was visualized to enter the lumen of the vessel. Group B was partially guided where ultrasound was used to identify the target vessel only. Finally, Group C received standard of care via the landmark approach.^5^ The third study by Stolz, et al was set in an ED and aimed to determine the number of attempts required to achieve proficiency with USGPIVA. The participants enrolled 796 patients and achieved an overall success of 88.24%.^18^ All of the participants were previously naïve to POCUS and the determinants of difficulty used in the study were not included in the report.

Assessment of difficulty of IV access varied considerably between the studies and was largely arbitrary. Most studies had an inclusion criterion of two failed blind attempts. Characteristics of difficulty included the patient reporting history of difficulty, inability to palpate a vessel, and significant comorbidities. Bahl, et al developed the most robust inclusion criteria, including: (1) the patient reports a history of “difficult stick;” (2) experienced at least one previous episode where two or more attempts were required to obtain a peripheral IV; and (3) at least one of the following: (a) prior history of a rescue catheter as a result of an inability to obtain a peripheral IV, (b) history of end-stage renal disease, (c) history of IV drug abuse, or (d) history of sickle cell disease.^20^

The approach to ultrasound technique was consistent throughout many of the studies. Eleven of the 16 reviewed studies taught a single operator, dynamic technique and encouraged participants to begin their attempt on the transverse short axis. Miles, et al observed nurse participants typically preferred the transverse approach initially, incorporating the longitudinal approach with more experience.^21^ Four of the studies didn’t describe the ultrasound approach they taught or used in the study. Price, et al utilized the transverse approach to measure vessels but didn’t describe the approach to catheterization.^22^

### Education and Training

The approach to training in the reviewed studies was significantly varied and ranged from 90 minutes to 20 hours. All of the training packages included a blend of didactic and hands-on learning, while only some required supervised attempts to assess proficiency. The educational approach of each study is summarized in Table 3.

**Table 3.** Outcome Measures and Ultrasonographic Approach

Study	# Patients/ Participants	Outcome Measure	First Stick Success (%) (USG)	Overall Success (%)	# Punctures	US Approach
Acuña, et al 2020^19^	483	• Success rate of USGPIVA placement • Complications associated with USGPIVA • Adequacy of handheld device for USGPIVA placement • Confidence level in performing USGPIVA with handheld device	84% First Attempt Success	92% Overall Success	N/A	In-plane 70% Out-of-plane 10% Not documented 20%
Ault, et al 2015^23^	8 Nurses (Patients Not Recorded)	• Number of USGPIVA placements that needed to be performed under supervision to achieve proficiency and consistency • Number of minutes required for successful vessel cannulation • Associated complications	N/A	N/A	N/A	N/A
Bahl, et al 2016^20^	124	• USGPIVA success rate • Time to USGPIVA placement	N/A	76% Overall Success	Mean USGPIVA: 1.52 per subject SOC: 1.71 per subject	N/A
Duran-Gehring, et al 2016^24^	830	• First attempt success USGPIVA • Overall PIV success • Number of blind punctures prior to USGPIVA	97.5% Overall Success	86.8% First Attempt Success	Mean SOC: 2.1 per subject USGPIC: N/A	Veins were examined in both transverse/long-axis planes to determine depth and width Single operator, transverse, out-of-plane approach for cannulation Rotate to long-axis to confirm position of catheter within lumen of the vessel
McCarthy, et al 2016^25^	1,617	• Success/failure on initial/second attempt • Occurrence of a complication • Patient reported pain associated with the procedure (0-10) • Duration of first attempt	82%-86% Regardless of Difficulty	80.9% Overall Success	N/A	Dynamic, single operator technique US utilized to visualize and guide the needle into the lumen
Oliveira, et al 2016^26^	65	• Success of physicians, nurses, and corpsmen utilizing USGPIVA • Number of attempts	Nurses: 63.2% Corpsmen: 50% Participants all novice with <5 USGPIVA procedures performed before study commencement	N/A	Average 2.8 per patient	Single operator, dynamic technique Participants encouraged to utilize transverse/longitudinal techniques A novel combination approach taught, involved participants inserting needle in transverse position then rotating probe longitudinally visualize the catheter in the vessel
Price, et al 2019^22^	100	• First attempt success rate between double tourniquet and single tourniquet groups (USGPIVA)	Single Tourniquet –79.2% Double Tourniquet – 76.5%	Single Tourniquet – 97.9% Double Tourniquet – 98%	Average 1 per patient (USGPIVA)	Participants measured vessels in short-axis orientation Approach to achieve cannulation was not reported
Resnick, et al 2008^27^	101	• Success of skin marking procedure (USGPIVA) • Procedural time • Perceived reason for blind failure • Target vein selection • Depth of target vein • Number of skin punctures • Length of catheter in the vein • Associated complications	59.6% First Attempt Success (Varying Experience)	73% Second Attempt	N/A	Target vessel identified; depth measured in short-axis Catheters were inserted using a dynamic, single operator technique All operators began the procedure in short-axis view and allowed to change to long-axis view if struggling to gain access
Salleras-Duran, et al 2016^4^	103	• Nurse perception of difficulty • Success rate USGPIVA overall/first attempt • Catheter longevity • Patient satisfaction	84.2% First Attempt Success	95.1% Overall Success	N/A	N/A
Schoenfeld, et al 2011^28^	219	• Success rate of USGPIVA • Complication rate • Rate of success based on previous ED technician experience with both standard approach and USGPIVA	78.5% First Attempt Success	Not Reported	Mean 1.35 (SD = 0.56)	Dynamic, single operator technique Both transverse/longitudinal methods were taught Participants encouraged to begin with transverse method
Shokoohi, et al 2013^9^	401,532	• Central venous catheter placement rate	N/A	N/A	N/A	N/A
Skulec, et al 2020^5^	300	• Compare first attempt success between three groups of varying approach • Compare overall success of cannulation • Number of attempts for successful cannulation • Time required to achieve cannulation • Prehospital complications	Fully USG technique where needle visualized to penetrate lumen (Group A) – 88% Partial USG technique visualizing target vessel only (Group B) – 94% Landmark approach (Group C) – 76%	Group A – 99% Group B – 99% Group C – 90%	Group A: 1.20 (SD = 0.57) Group B: 1.07 (SD = 0.29) Group C: 1.45 (SD = 0.90) P <.001	Scanning with transverse probe orientation to identify target vein Compression test to differentiate between vein and artery Color doppler was used optionally by the operator Participants instructed to preferentially use transverse approach
Stolz, et al 2016^18^	796	• Number of attempts required to achieve proficiency and consistency • Overall success rate • Determinants of difficulty	N/A	88.24% Overall Success	N/A	In-plane, longitudinal approach where needle was guided into the vessel was emphasized for PIV access Participants familiarized with color doppler, compression technique, and transverse method
Vinograd, et al 2018^29^	58 (300 USGPIVA Attempts)	• First attempt success • Complication rates • USPGIV longevity	68% First Attempt Success	91% Overall Success	N/A	All PIVs were placed using the dynamic method in the short-axis
Weiner, et al 2013^10^	50	• Rate of physician intervention • Mean time to PIV placement • Number of skin punctures • Patient satisfaction • Patient perception of pain on 10-point scale	N/A	N/A	Mean: 2	Dynamic, single operator technique Nurses were instructed to use the transverse approach at 45° oblique angle to the vessel
Miles. et al 2012^21^	9 Initial Participants	• Rate of physician intervention • Time to PIV placement • Number of skin punctures • Patient satisfaction • Patient perception of pain	N/A	N/A	N/A	Nurses were taught both transverse/longitudinal approaches Participants typically preferred transverse method until more experienced

Abbreviations: US, ultrasound; USG, ultrasound-guided; USGPIVA, ultrasound-guided peripheral intravenous access; PIV, peripheral IV; ED, emergency department; SOC, standard of care.

## Discussion

This scoping review examined 16 articles to identify the utility of non-physician USGPIVA in all settings. Currently, POCUS is an emerging diagnostic adjunct in non-physician clinical care, especially for out-of-hospital providers.^16^ Ultrasound technology has advanced to facilitate smaller, more portable, and cost-effective devices that can be translated to non-physician practice and can potentially provide both diagnostic and therapeutic advantages.^4,19^

Peripheral IV access is one of the most commonly performed skills by paramedics and nurses in both the out-of-hospital and in-hospital environments.^1–3^ Difficulty achieving PIVA is frequently encountered and alternative methods must be sought to establish venous access.^13,25^ This often requires the input of a more senior clinician or physician.^9,10,24^ Existing physician-based literature on this topic, not included in this review, expounds the advantages of ultrasound-guided technique in improving success, reducing number of punctures, reducing time of procedure, and improving patient satisfaction.^14^ Ultrasound-guided PIVA is routinely performed by physicians in the ED; however, emerging literature suggests nurses, paramedics, and ED technicians can competently perform this skill with relatively little additional training.^18,23^

The participants included in the studies were of mixed background and experience. Cohorts included nurses, paramedics, emergency technicians, and military corpsmen with experience ranging one year to thirty-five years. Previous exposure to ultrasound was varied and many of the participants were ultrasound naïve and were provided with training as part of the study. Ultrasound-guided PIVA is a well-established practice in EDs globally and is typically carried out by emergency physicians to gain peripheral or central vascular access in patients that have been failed by the traditional method.^9,14^ Increasingly, this practice has been studied for adaptation to the scope of other health care providers in the emergency setting.^5,6,16,20^ All providers in the studies were already proficient in the traditional method of PIVA; therefore, the ultrasound-guided technique represented an extension of an existing skill. The literature suggests that non-physician health care providers can capably perform USGPIVA with minimal training and supervision.

The approach to training participants was non-standardized and ranged from 90 minutes to 20 hours. Three of the studies aimed to evaluate the learning curve associated with training nurses, paramedics, and ED technicians in USGPIVA.^18,23,24^ The majority of studies had a training duration of two hours, with some outliers, and this appears to be sufficient to engender proficiency. Duran-Gehring, et al reported that a cohort of 830 ED technicians achieved an USGPIVA rate of 97.5% after completing a brief but comprehensive training program.^24^ Training programs typically included a blend of didactic teaching, hands-on simulation, and supervised practice on live patients. Stolz, et al sought to define the learning curve and determined a positive correlation between number of attempts and participant proficiency. Nurses and paramedics achieved a success rate of 88% after 15-26 attempts.^18^ A confounding variable identified in many of the studies was significant inconsistencies amongst participant experience where some participants were highly experienced veterans while others only had one year of experience.^26,28^ A review appraising educational standards for paramedic POCUS suggests “paramedics may be able to gain proficiency in POCUS reasonably promptly, regardless of base qualification, experience, duration, or perceived quality of training.”^16^ These studies conclude that with relatively minimal, but comprehensive training, non-physicians can become proficient and improve success in USGPIVA with experience.

Determining DIVA appeared arbitrary in many of the studies with one study relying on a discretionary approach based on perceived difficulty and failed blind attempts.^19^ Some studies developed a criterion for inclusion made up of characteristics known to increase difficulty (ie, obesity, IV drug abuse, and multiple comorbidities) while others didn’t document the method they used to determine difficulty.^20^ Ultimately, there is a lack of consensus as to what defines “difficult” PIVA, making comparison between studies and patient populations difficult.^19,25^ This study revealed an externally validated scale predictive of difficult PIVA in adults (A-DIVA) that may help standardize the approach in determining difficult PIVA.^13^ The modified A-DIVA tool developed by van Loon, et al resulted from a large, multi-center, prospective study that enrolled 3,587 patients who failed first attempt peripheral venous access. The resultant data were analyzed and a five-variable additive A-DIVA scale was created based on patient characteristics that affect the outcome of peripheral IV cannulation on first attempt.^13^ This externally validated assessment tool appears reliable, generalizable, and predictive of adults at risk of DIVA.^13^ Utilization of the A-DIVA scale as a meaningful, quantitative metric can potentially standardize the approach to difficult PIVA as opposed to relying on experience or operator gestalt.^13^

This scoping review suggests there are clinical implications to the introduction of non-physician USGPIVA. Typical practice in both ED and out-of-hospital is for non-physician providers to establish PIVA through the landmark approach.^20^ If difficulty is encountered or anticipated, the provider may make a blind attempt or escalate to a more senior clinician or physician.^9,10^ Ultimately, if peripheral venous access is unable to be achieved, the patient may require CVC placement in the ED or IO access out-of-hospital as an alternative. Placement of a CVC is associated with a greater risk profile of blood stream infection, pneumothorax, and large artery cannulation, which therefore is undesirable for patients who don’t specifically require central venous access.^10,30^ Shokoohi, et al assessed the rate of CVC placement in ED patients over a six-year study period after the implementation of an USGPIVA program.^9^ This study saw a reduction in CVC placement by up to 80%, especially in the non-critically ill population.^9^ In addition to potentially increased risk, the process of having to escalate to a more senior clinician to facilitate vascular access both delays intervention and is a resource burden.^10^ Weiner, et al postulated that appropriately trained emergency nurses could reduce the need for physician intervention in patients with difficult vascular access. Their study discovered that in patients assigned to standard of care (landmark approach), physicians were required to intervene in 52.4% of cases, whereas they were only in 24.1% of cases assigned to an ultrasound-guided technique.^10^ These studies were the only two that specifically investigated the implications associated with introduction of a non-physician-led, ultrasound-guided IV access program and both reported favorable outcomes.

While some of the study cohorts included paramedics, only one was exclusive to the out-of-hospital environment.^5^ There is an apparent dearth of literature evaluating USGPIVA placement in the out-of-hospital environment. The existing body of literature is largely supportive of non-physician USGPIVA in-hospital, and given the broad similarities between the professions, should be translatable to the out-of-hospital environment.

## Recommendations

The clinical definition of “difficult” IV access remains arbitrary and non-standardized. Literature exploring the characteristics associated with DIVA exists, and there has been movement toward the creation of a validated assessment scale that could be utilized to predict DIVA in adult patients. Further investigation into the value of USGPIVA for non-physician providers would benefit from a standardized definition of DIVA.

The clinical application of USGPIVA in the in-hospital setting is reasonably well-demonstrated with a growing body of evidence supporting implementation of non-physician-based USGPIVA. Literature examining the application of this practice with both a handheld POCUS device and paramedics in the out-of-hospital environment is scarce. This review identified only one study of such a design.

A large, randomized, controlled trial incorporating a standardized DIVA tool with non-physician providers in the out-of-hospital environment would be valuable to broaden the scope of USGPIVA and measure paramedic proficiency. The study would ideally consider first attempt success, overall success, USGPIVA versus landmark method, time to achieve PIVA, number of skin punctures, operator experience, and any associated complications.

## Limitations

The authors acknowledge the limitations of the scoping review methodology. The articles recovered were generally heterogenous in study design and of low to medium quality. Authors SB, JD, BM, and SJ are all operational paramedics and BM performs USGPIVA in clinical practice. Therefore, there is an acknowledged risk of bias in article selection and interpretation.

## Conclusion

Ultrasound-guided PIVA for non-physician health care providers appears to be a feasible and effective extension to already established practice. Nurses, paramedics, and ED technicians appear to be able to achieve proficiency, consistency, and a high degree of success when learning and performing USGPIVA. Variations in success were accounted for by variations in experience, which was demonstrated to improve with on-going acquired experience. The lack of a standardized DIVA assessment tool makes it difficult to reliably compare studies. Very little literature exists exploring the feasibility and success of paramedics performing USGPIVA in the out-of-hospital environment. Further studies incorporating a standardized DIVA assessment tool and set in the out-of-hospital environment would aid in validating the clinical utility for POCUS and USGPIVA.
